# Identifying and Evaluating the Essential Factors Affecting the Incidence of Site Accidents Caused by Human Errors in Industrial Parks Construction Projects

**DOI:** 10.3390/ijerph191610209

**Published:** 2022-08-17

**Authors:** Adel Rafieyan, Hadi Sarvari, Daniel W. M. Chan

**Affiliations:** 1 Department of Civil Engineering, Najafabad Branch, Islamic Azad University, Najafabad 85141-43131, Iran; 2 Department of Civil Engineering, Isfahan (Khorasgan) Branch, Islamic Azad University, Isfahan 81551-39998, Iran; 3 Department of Building and Real Estate, The Hong Kong Polytechnic University, Hung Hom, Kowloon, Hong Kong, China

**Keywords:** construction projects, construction accidents, human error, industrial area, fuzzy Delphi method, SWARA

## Abstract

In terms of safety management, the implementation of industrial parks construction projects (IPCPs) is incredibly challenging due to the special working conditions and the specific type of use of the buildings. On the other hand, the possibility of causing accidents in these areas based on human errors is high and important for project execution due to the risks of human errors and financial losses. Therefore, this study tries to fill this existing research gap by identifying and evaluating the effective key factors leading to the occurrence of construction accidents caused by human errors in the development of IPCPs. After a holistic review of the reported literature, four rounds of fuzzy Delphi survey were launched to capture the individual opinions and feedback from various project experts. Accordingly, 41 key factors affecting human errors in the implementation of industrial parks construction projects in Iran were identified and classified into nine main groups of wrong actions, observations/interpretations, planning/processes, equipment, organization, individual activities, environmental conditions, rescue, and technology. Then, the step-wise weight assessment ratio analysis (SWARA) method was adopted to rate and rank the identified factors of human errors in the implementation of IPCPs in Iran. The research findings indicated that among the elicited factors, time factor (0.1226), delayed interpretation (0.1080), and incorrect diagnosis/prediction (0.0990) are the three most crucial factors leading to human errors in the implementation of IPCPs in Iran. The results of this research study have provided various major project stakeholders with an effective decision-aid tool to make better-informed decisions in managing and reducing the occurrence of construction site accidents particularly caused by human errors associated with IPCPs.

## 1. Introduction

The construction industry is one of the most dangerous industries in terms of work-related losses, injury rates, and compensation to workers. In this industry, injuries leading to death, serious occupational injuries, and lost work time occur due to its unique nature, and a high number of accidents cause severe human and financial damage to communities [1]. Continuous changes in the work environment, use of various resources and tools, unsuitable working conditions, unsustainable employment, and unsuitable working environments are among the characteristics of the construction industry causing accidents [2]. Some accidents cause physical damage and destruction of part of the project, which will hurt the work efficiency of other project staff [3]. In this regard, paying attention to the observance of safety principles and prevention of accidents and diseases caused by work in workshops has a high priority in projects, especially large projects [4]. Therefore, there are no shortcomings in terms of legal principles, but in general, the statistics of work-related accidents in Iran show an unacceptable increase compared to developed countries. According to reports, construction project accidents in Iran are ranked higher compared to global scales [3]. Now, according to the evaluation and research, it has been determined that most accidents are caused by negligence and non-observance of safety principles in the use of inappropriate machinery and equipment. Studies show that one of the main causes of accidents in construction projects is human error [5,6].

Construction projects involve remarkably diverse and complex activities, so there are many risks in such projects. Of course, these risks vary according to the conditions and environment of construction projects. For example, project risks and threats are different in urban and industrial environments [4]. Naturally, the implementation of IPCPs is associated with more complexities. Artisans and their social issues and expectations and influential groups that are formally or informally sensitive about projects or their consequences are among the issues that complicate projects and the consequences of accidents. Physical space limitation, the activity of industrial units, the high volume of light and heavy machinery traffic, and the environment in which the project is implemented increase the complexity of implementation and accident prevention and its risk. Therefore, the effects and consequences of non-observance of safety and accidents of human error are very costly for stakeholders and sometimes will be irreparable. In this regard, drawing on the experiences of previous projects is one of the most important measures to be taken in large construction projects in industrial environments [5]. By reducing and controlling human error, project risks and hazards can be reduced [6]. The purpose of learning from human error is to identify the source of risks and uncertainties, and their effects and provide an appropriate management response to these risks. Effective risk management includes four processes: risk identification, risk assessment, risk response, and risk assessment and monitoring [7]. The purpose of these processes is to minimize the effects of risks on project objectives by eliminating or sharing risks.

A review of the research literature indicates that extensive studies have been conducted in the fields related to human errors and the factors affecting them in urban construction projects [5,6,7]. While the research literature shows few studies on the factors affecting human errors in industrial environments and areas in Iran, the execution of IPCPs is incredibly challenging due to the special working conditions and the specific type of building use [8]. Therefore, this study aims to identify and evaluate the essential factors affecting the occurrence of site accidents caused by human errors in the development IPCPs in the developing country of Iran and attempts to fill the extant knowledge gap between previous studies. Identifying the factors affecting the accidents caused by human errors can play an effective role in project management decision making. The input parameters for evaluating accidents caused by human errors are not accurate and are subject to great uncertainty and are not considered in various types of research [1,2,5]. In this research, the fuzzy Delphi survey technique was used to screen the factors to apply the uncertainty of experts’ opinions. The innovation of this research is the application of the fuzzy Delphi survey technique in reducing uncertainty and identifying the effective factors of human errors and the use of the SWARA method for prioritization, which reveals the various dimensions of human errors in IPCPs based in Iran. Therefore, the main question of the present study is as follows: what is the importance of the effective factors in the occurrence of construction accidents caused by human errors in the implementation of IPCPs? To answer the stated research questions, first of all, the international research literature was comprehensively reviewed to accurately identify the key factors affecting the events caused by human errors. Then, using the Delphi survey technique in four different rounds, the factors extracted from the research literature on IPCPs in Iran were reviewed and screened. Then, the list of final identified factors were evaluated using the SWARA method, and the most crucial factors were determined. The results of this study can help various major project stakeholders as decision facilitators to make better decisions in managing or reducing human errors associated with construction site accidents.

## 2. Research Background

### 2.1. Human Errors in the Construction Industry

Human error is part of our daily experience [9]. Occupational accidents have killed more than 300,000 people and injured more than 300 million worldwide each year [10]. According to researchers, the cost of occupational accidents in the construction industry may be up to 15% of the total cost [11]. Workplace safety is a major concern in many countries. Among the various industries, the construction sector is known as the most dangerous workplace. Construction accidents not only cause human suffering but also lead to a great deal of financial loss. To prevent the recurrence of similar incidents in the future and to prepare scientific risk-control programs, incident analysis is essential [7].

Studies of human factors show that the root of about 80% of major accidents affecting safety and environment or ergonomics is human error [2]. Many attempts have been made to define and classify human error. For example, Tixier et. al. [12] defined an error as an unauthorized act when the permissible operating limits are defined by the system. One of the most important classifications that has been accepted as a suitable model for describing human function since its introduction is the model proposed by Riesen in 1990. He proposed an error-modeling system based on the classification of skill, knowledge, and rule-based behavior [13]. Human error must be considered beyond tangible events. Errors in economic planning or military orders should also be investigated and analyzed in their place and depending on the intended purpose [14].

Sudani [15] stated that many events occur in the world every year. Some of these accidents lead to damage to the environment, and others lead to harm to humans. Environmental disasters, such as the release of various pollutants, affect water, soil, and air. Occupational accidents occurring due to non-compliance with the principles of health and safety can threaten the health of people, cause disability, and even in acute cases lead to death. Accidents are usually the result of unsafe conditions or unsafe acts. In general, financial or human losses are the negative consequences of industrial accidents.

The consequences of a human error can be from minor to very severe; in addition, they may vary from one situation to another, from one job to another, or from one piece of equipment to another. Concerning equipment, the consequences of human error may fall into three categories: (i) equipment operation ceases completely; (ii) the equipment does not stop working completely; and (iii) equipment performance delays are negligible. Human errors in engineering can be classified under different classifications. The seven common classifications are as follows: (1) maintenance errors; (2) operator errors; (3) design errors; (4) assembly errors; (5) inspection errors; (6) error management; and (7) participatory errors [16,17].

### 2.2. Factors Affecting Human Errors in the Construction Industry

Over the years, various researchers in the field of human engineering have stated that there are many general factors that significantly increase stress on the individual and, in turn, lead to a significant deterioration in his reliability [16,18,19]. Some of these general factors are as follows: poor health, the possibility of redundancy at work, having to work with people with unpredictable moods, serious financial problems, working under very high pressures and not having the right expertise to do the work in progress, experiencing problems with a spouse or children or both, poor chances for promotion, and excessive demands on people in the workplace [17]. Experience shows that there are many reasons for human error. Some critical issues are poor training, poor equipment design, poor motivation, complex work, poor equipment operation and maintenance methods, insufficient workplace lighting, poor management, etc. [20,21,22].

In another study, Azhdari [23] investigated the causes of accidents caused by human error in maintenance operations in the petrochemical industry. They identified and documented nineteen different causes of human error. They categorized the identified factors into four levels: unsafe practices, unsafe supervision, preconditions for unsafe practices, and organizational effects. They acknowledged that increasing the effectiveness of staff training and improving monitoring of staff performance have the greatest role in reducing the incidence of human error in petrochemical maintenance operations, respectively. Similarly, Jahani [2] classified human errors leading to accidents in one of the cement factories into four categories. The results of this study show that most errors are from the first level, i.e., errors due to unsafe acts. They also cited error-based error, poor industrial environment, inadequate monitoring, and poor resource management as important causes of error.

Chan et al. [24] considered the lack of strong safety culture as the main reason for many accidents. Mohajeri et al. [25] examined the four criteria of cost, quality, time, safety, and ergonomics to evaluate human errors in the implementation of the construction project with emphasis on the principles of ergonomics. They identified and assessed a total of 20 risks of error. Salimi [26] also acknowledged that the implementation of construction projects is always affected by many dangers, such as falls on people and equipment, injuries, burns, electric shocks, accidents, falls, etc. He stated that the incidence of these accidents in Iran is about three times the global average, and often, the consequences of these accidents are very heavy for projects.

Morais et al. [27] studied the analysis of human reliability of human actions and external factors through the project life cycle. They cited factors such as inadequate skills, insufficient information, inadequate quality control, inadequate communication, inadequate working hours, design problems, management issues, social pressures, and inadequate task allocation as factors in human error. Shi et al. [28] stated that due to the nature of construction activities, construction workers usually work in partnership; therefore, interpersonal influences among workers play an essential role in shaping and influencing the safety behaviors of construction workers. Amiri et al. [10] examined occupational accidents in road construction projects. They cited factors such as improper driving of road construction machinery, burnout, heat, poor hygiene, and collision with machinery as the most important causes of accidents in this type of project. Xu et al. [29] reviewed the development of an incident learning model to assess the ability of construction workers during safety training. Improving the safety performance of construction workers lies strongly in the safety training and training of technologies, materials, and organizations. The results of their research also showed that age, experience, business, type of project, type of organization, and site environment affect the characteristics and learning abilities of workers, which leads to various levels of safety perception, awareness, and performance. Dhalmahapatra et al. [30] investigated the causes of crane-related accidents. Cranes serve in the construction industry and are used to transport materials in complex work environments. They stated that the complexity involved in machine-human interaction in the workplace puts it at risk. They also acknowledged that the number of accidents occurring during construction and maintenance activities increased over the weekend.

A review of the research literature shows that although many studies have been conducted on human error, a comprehensive list of factors affecting the occurrence of human error in the construction industry, especially in industrial areas, is not available. Therefore, to fill the gap in previous studies, this study seeks to determine the effective factors in the occurrence of accidents caused by human error in the development IPCPs. In this regard, based on a comprehensive review of the research literature, 54 effective factors in the incidence of human error were identified and categorized in 17 groups, which were used as the first-round questionnaire of the Delphi technique (Table 1).

## 3. Research Methodology

The present study was conducted to identify and rank the factors affecting the incidence of accidents caused by human error in the development IPCPs. For this purpose, the effective factors in the occurrence of accidents caused by human error were first studied through the literature, and the list of factors was then reinforced using the four-round fuzzy Delphi survey method already used for similar research studies [34]. Then, the SWARA method was used to rank the factors.

### 3.1. Fuzzy Delphi Survey Method

In this study, Delphi panel members consisted of 15 experts with more than 20 years of experience in the HSEE (Health, Safety, Environment, Energy) sector of IPCPs. The research literature on how to select and hire specialists who respond to the Delphi questionnaire does not show any strong and explicit rules. However, it should be noted that the quality of specialists is more important than their number and quantity [34]. Hence, participants in the Delphi survey of experts and critics who must have sufficient knowledge and experience in a similar subject should have sufficient time to participate and effective communication skills [18]. In terms of the number of specialists involved, this is usually less than 50 and often from 10 to 20 [35].

Ishikawa et al. [36] acknowledged that in implementing the Delphi method, it is better to obtain data in a natural language from specialists and analyze the data using fuzzy sets. The advantage of the fuzzy Delphi method is that it considers each of the ideas and combines them to reach a group agreement [14,37]. The implementation steps of this method are a combination of the traditional Delphi method and the analysis of the data of each step using the definitions of fuzzy set theory. The steps of the fuzzy Delphi method in the present study are as follows: (1) identification of research indicators using a comprehensive review of theoretical foundations of research; (2) collection of the opinions of decision-making experts; (3) verification and screening of indicators; and (4) consensus and completion of fuzzy Delphi.

Based on previous studies and initial monitoring by researchers, the first-stage Delphi questionnaire including 54 factors affecting the incidence of human error accidents was developed in 17 groups. The steps were such that to determine that the identified factors can be considered effective factors in the occurrence of accidents caused by human error in the industrial areas of Iran, 15 experts were asked to comment. The results of the first round indicated that out of 54 factors, 5 factors were removed from the questionnaire, and 2 factors were added. In the second round, a new questionnaire with 51 factors was sent to the experts. In this round, 42 factors had the necessary validity, but it was necessary to combine 2 factors with other similar cases and with close meanings. Thus, a new questionnaire with 40 factors was sent again to the experts in the third round. In this round, 38 factors showed the necessary validity, and based on the new theories of experts, 3 items were added. In the fourth round, 41 factors were re-sent to the experts, and then, at this stage, all experts concluded that all 41 identified factors could be identified as effective factors in the occurrence of accidents caused by human error in Iran’s industrial areas. The 41 identified factors were classified into 9 groups: misconduct, observations/interpretations, planning/processes, equipment, organization, individual activities, environmental conditions, rescue, and technology. The final factors were examined and confirmed based on face, content, and structural validity (Table 2). Factors can be added and removed based on fuzzy Delphi results. The ultimate goal of adopting the fuzzy Delphi technique is to identify the essential key factors that adequately answer the research questions. The face validity of the questionnaire was confirmed based on the opinions of several respondents, whereas the construct validity of the questionnaire was warranted using the SmartPLS software.

### 3.2. Step-Wise Weight Assessment Ratio Analysis (SWARA) Method

Based on the results of the Delphi stage, 41 factors in nine different groups were determined for the final evaluation. Then, the SWARA method was used to prioritize the identified factors. In this study, the purposeful sampling method was used to select the respondents to the survey, which was conducted by other researchers for similar research questions [11]. According to the subject of the study, the statistical population includes 220 engineers and construction management specialists, insurance experts, and HSE experts of industrial park company. In the present study, the sample size was calculated using Cochran’s sample size formula [38,39]. Based the Cochran’s sample size formula, 140 people were considered as the number of statistical samples of the entire population, and the same number of questionnaires were distributed, of which 136 questionnaires were returned. The return rate of the questionnaire was 97.14%. The available sampling method was used for sampling.

SWARA is one of the new multi-criteria decision-making methods introduced by Violeta [38]. This method is used to calculate the weight of the criteria. In the SWARA method, experts first arrange the criteria in order of importance. The most important criterion is placed first and obtains a score of one. Finally, the criteria are ranked based on the average values of relative importance. The steps of SWARA method are as follows.

Step 1: Sort the criteria: First, the criteria are written in order of importance. The most important criteria are at the higher ranks, and the less important criteria are at the lower ranks.

Step 2: Determine the relative importance of each criterion (*S_i_*): In this step, the relative importance of each criterion to the previous criteria is determined. In the SWARA method process, this value is denoted by *S_i_*.

Step 3: Calculate the *K_i_* coefficient: The *K_i_* coefficient, which is a function of the relative importance of each criterion, is calculated using Equation (1):

*K_i_* = *S_i_* + 1 (1)

Step 4: Calculate the initial weight of each criterion: The initial weight of the criteria is calculated through Equation (2), where it should be noted that the weight of the first criterion, which is the most important criterion, is considered equal to 1.

*Q_i_* = *Q_i_* − 1/*K_i_* (2)

Step 5: Calculate the final normal weight: In the last step of the SWARA method, the final weight of the indicators, which is also considered the normalized weight, is calculated through Equation (3). Normalization is performed in a simple linear method.

*W_i_* = *Q_i_* ÷ (∑*Q_i_*) (3)

As mentioned, the main feature of the SWARA method is that it is possible to evaluate the opinions of experts or assessment teams about the importance of indicators in the process of determining their weight [38].

## 4. Presentation of Analytical Results

In this study, the factors affecting human errors were prioritized by the SWARA method. In the SWARA method, experts first arrange the criteria in order of importance. The most important criterion is placed first and obtains a score of one. Finally, human error indices are ranked based on the mean values of relative importance. First, the indicators of human errors were sorted by importance. Then, the relative importance of each criterion was determined compared to the previous criteria. These values are listed in the “Mean Relative Importance” column in Table 3, which is (*S_i_*). In the next step, the coefficient (*K_i_*) was calculated. The coefficient (*K_i_*) is one for the time index (*S*_01_), which is the most important. This value was calculated for other indicators of human errors. To calculate the initial weight of each criterion, Formula (2) was used. For example, the values *Q*_1_, *Q*_2_, and *Q*_3:_

$$Q_{1}=1$$

$$Q_{2}=\frac{Q_{1}}{K_{2}}=\frac{1}{1.13}=0.885$$

$$Q_{3}=\frac{Q_{2}}{K_{3}}=\frac{0.885}{1.08}=0.819$$

**Figure 1:**
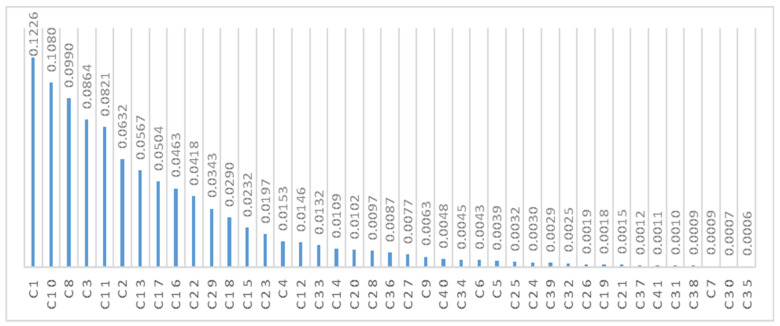
The final weight of human error indicators by the SWARA method.

These values are presented in the “Initial Weight” column in Table 3. To calculate the final weight, the linear normalization method was used according to the Equation (3). Thus, as shown in Table 3 and Figure 1, the final weight of each factor was obtained.

According to the obtained results, it was found that the time factor (C_1_) with a weight of 0.1226 is the top priority factor. Furthermore, factors of delayed interpretation (C_10_) with the weight of 0.1080, misdiagnosis/prediction (C_8_) weighing 0.09900, instrumental (C_3_) weighing 0.0864, lack of necessary controls (C_11_) weighing 0.08210, functional (C_02_) weighing 0.0632, prioritization/scheduling error (C_13_) weighing 0.0567, equipment fraction (C_17_) weighing 0.0504, software error (C_16_) weighing 0.0463, and lack of training (C_22_) weighing 0.0418 are in the second to tenth priorities, respectively. On the other hand, factors of unfamiliarity with technology (C_41_) weighing 0.0011, inappropriate temperature (C_31_) weighing 0.0010, lack of safety equipment (C_38_) weighing 0.0009, incorrect reasoning (C_7_) weighing 0.0009, improper learning (C_30_) with a weight of 0.0007, and non-implementation of fire alarm system (C_35_) with a weight of 0.0006, respectively, have the least impact on the occurrence of site accidents caused by human errors in the development IPCPs.

## 5. Discussion of Survey Results

The innovation of this research is the combination of fuzzy Delphi survey technique and the SWARA method for human error analysis with uncertainty reduction. The results of this study are relatively consistent with the results of previous studies. For example, Azhdari [23] reached the conclusion that increasing the effectiveness of staff training and improving employee performance monitoring play a key role in reducing the incidence of human error events in the industry. Morais et al. [27] introduced factors such as inadequate skills, lack of sufficient information, inadequate quality control, inadequate communication, inadequate working hours, design problems, and management issues as causes of human error. Amiri et al. [10] also found that factors such as burnout and heat are considered as serious factors in the occurrence of accidents in construction industry projects. Xu et al. [29] acknowledged that reducing the risks of human error among construction workers lies strongly in the issue of education. Their research also showed that age, experience, and site environment are influential in the occurrence of accidents. Dhalmahapatra et al. [30] also considered the issue of lack of proper interaction between humans and technology in the occurrence of accidents caused by human errors.

Considering the results of previous research and the results obtained in the present study, it can be pointed out that in recent years, many efforts have been made by researchers to reduce human error in the construction industry. However, the key stakeholders of construction projects (employers and contractors) still face many unknown ambiguities and factors to improve safety in the construction industry. At the same time, increasing safety in construction, which is one of the main factors in the success of projects, is a vital issue in the development of the construction industry. In this regard, a thorough study of the factors affecting the incidence of human error, especially in developing countries, is essential. Focusing on factors such as wrong actions, observations/interpretations, planning/processes, equipment, organizing, individual activities, environmental conditions, rescue, and technology can be a positive step in reducing gaps and problems related to safety in the construction process.

As the results of the present study show, in the group of wrong actions, time and instrumental and performance factors are the most effective factors in this group in the occurrence of accidents caused by human error in the construction industry. In the group of observations/interpretations, delayed interpretation, incorrect diagnosis/prediction, and lack of necessary controls are the most important causes of accidents. In the planning/processes group, prioritization/scheduling error, improper design, and improper construction methods are also critical factors. In the equipment group, the most crucial factors in the occurrence of accidents are equipment shortages, software errors and equipment failure, as well as lack of training, inadequate organizational charts, and inadequate working hours are also the most important factors in the organizing group. In the group of individual activities, fatigue, workplace diversity, and carelessness are the most effective cases. In the group of environmental conditions, the influential factors are inadequate humidity, inadequate lighting, and inadequate sound. Lack of firefighting and non-deployment of emergency medical teams are also crucial factors in the rescue team. Finally, in the technology group, the factors of technology incompatibility with the existing conditions and excessive confidence in technology are considered key effective factors.

Future research could enhance the generalizability of the proposed results by increasing the number of construction professionals to evaluate in a similar study. However, as suggested by Sarvari et al. [39], it will also be valuable to compare the factors affecting the incidence of human errors according to the level of development of the studied countries (developed versus developing) to identify any similarities and differences. The results of this study can offer various key project stakeholders a useful decision facilitator to make better-informed decisions in managing and reducing the occurrence of construction site accidents particularly caused by human errors. Finally, artificial intelligence and deep learning can be useful in human error analysis due to their high ability to solve various scientific problems [40,41,42].

## 6. Conclusions and Research Implications

This study aimed to identify and evaluate the key factors affecting the incidence of human errors in IPCPs in Iran. For this purpose, the effective factors in the occurrence of human errors were extracted by reviewing the research literature and then monitored by performing four rounds of fuzzy Delphi survey. Finally, 41 important and influential factors were reviewed and identified. The researcher-made questionnaire was compiled based on 41 factors identified under 9 main groups, including wrong actions, observations/interpretations, planning/processes, equipment, organization, individual activities, environmental conditions, rescue, and technology, based on a 5-point Likert scale of measurement. The face, construct validity, and also reliability of the questionnaire was examined and confirmed. Then, a self-administered survey questionnaire was distributed among the experts. Based on the Cochran sample size formula and using the available sampling method, 136 construction experts in Iran were selected as a statistical sample for the study. After collecting the returned questionnaires, the SWARA method was applied to analyze the opinions, and feedback was gleaned. Accordingly, in the ranking of groups, groups of wrong actions, observations and interpretations, equipment, planning/processes, organization, individual activities, environmental conditions, rescue, and technology were ranked from 1 to 9, respectively. Furthermore, in ranking the identified factors by considering all groups, time factor, delayed interpretation, incorrect diagnosis/forecasting, instrumental, and lack of necessary controls respectively were identified as the five most influential factors leading to the occurrence of human errors in the implementation of IPCPs in Iran. In this research, the time factor had the most weight in the human error analysis, so the project manager’s goal is to complete the project on time, and various potential risks have caused the project to be delayed, which increases the delivery speed of the project and increases human errors as well. The time goal and safety goal in projects should be optimized so that both safety and time are well-balanced and do not harm each other.

In terms of practical implications related to the results of this study, to reduce human errors in the construction industry, especially in developing countries, it is recommended to determine the relevant crucial factors in advance and also to set industry standards and protocols for organizations. A structured definition of safety management dramatically increases the chance of reducing and controlling human errors in the construction industry. In practice, it is essential to use the fuzzy multi-criteria decision-making method to analyze the human errors in a dynamic way so that, in the first stage, questionnaires are completed at monthly intervals. In the second stage of the project, the survey data are entered into the software, and in the third stage, the human errors are reviewed and analyzed, and the necessary improvement actions or mitigation strategies can be executed.

In terms of theoretical concepts, this study helps to better manage site safety in construction projects by identifying the key factors influencing the incidence of human errors in the implementation of IPCPs from a quantitative perspective, which has not been studied before, according to the authors’ knowledge and belief. In particular, it demonstrates that it is the control of people and equipment actions as well as the proper planning of various construction processes that allow construction companies to help improve their effectiveness and efficiency in site safety management. To succeed in safety management, construction companies may need proper organization, control of individual activities, improvement of environmental conditions, updates of the organization in terms of technology advancement, and increase in the facilities and equipment of rescue in their companies. Hence, some of the future research guidelines for widening and deepening the generated survey findings are as follows: What are the specific management and environmental capabilities that will allow construction companies to achieve better performance in the field of site safety? How can technology development contribute to the success of construction companies in improving site safety? What are the possible human error-mitigation strategies that can be adopted by construction companies for strategic site safety performance improvement?

## Figures and Tables

**Table 1:** Key factors contributing to human errors based on the review of the reported literature.

No.	Groups	Factors	References
1	Act at the wrong time	Timing	[14,31,32]
2		Duration	[32,33]
3	The action of the wrong type	Force	[32,33]
4		Space	[14,31,32]
5		Speed	[14,31,32]
6		Direction	[32,33]
7	Acting on the wrong equipment	Wrong equipment	[14,31,32]
8	Action in the wrong place	Sequence	[14,31,32]
9	Observation	Missing observation	[14,31,32]
10		Wrong view	[32,33]
11		Misdiagnosis	[32,33]
12	Interpretation	Error detection	[27,32,33]
13		Wrong argument	[32,33]
14		Decision error	[32,33]
15		Delayed interpretation	[32,33]
16		Incorrect prediction	[32,33]
17	Planning	Incomplete design	[27,32,33]
18		Prioritization error	[32,33]
19	Temporary people in the project	Error retaining information	[32,33]
20		Fear	[27,32,33]
21		Distractions	[27,32,33]
22		Fatigue	[27,32,33]
23		Work variety	[27,32,33]
24		Neglect	[27,32,33]
25		Stress	[27,32,33]
26		Physiological	[14,31,32]
27	Permanent people in the project	Functional defects	[14,31,32]
28		Improper learning	[14,31,32]
29		Tendency to think in a certain way	[14,31,32]
30	Equipment failure	Hardware failure	[14,31,32]
31		Software failure	[27,32,33]
32	Processes	Improper construction method	[32,33]
33	Information issues	Access to information	[32,33]
34		Vague information	[27,32,33]
35		Incomplete information	[27,32,33]
36	Communications	Incomplete communication	[32,33]
37		Communication failure	[32,33]
38	Organizing	Failure to organize	[32,33]
39		Improper quality control	[32,33]
40		Management problem	[27,32,33]
41		Design failure	[27,32,33]
42		Social pressure	[27,32,33]
43	Training	Insufficient skills	[27,32,33]
44		Insufficient knowledge	[32,33]
45	Environmental conditions	Improper temperature	[32,33]
46		Inappropriate sound	[27,32,33]
47		Unfavorable weather	[27,32,33]
48		Inadequate lighting	[27,32,33]
49		Undesirable humidity	[27,32,33]
50		Adverse environmental conditions	[27,32,33]
51	Work conditions	Type of employment	[14,31,32]
52		Irregular working hours	[14,31,32]
53		Inadequate team support	[27,32,33]
54		Improper work design	[32,33]

**Table 2:** Results of the fourth phase of the fuzzy Delphi survey for the key factors affecting the occurrence of construction accidents in industrial parks construction projects.

No.	Groups	Factors	Descriptions/Definitions
1	Wrong actions	Time	Wrong time action/wrong time allocation.
2		Operational	Lack of attention to the observance of priority and delay in the implementation stages.
3		Tools	Using inappropriate tools to perform executive operations.
4		Place	Performing operations in the wrong place.
5	Observations/ interpretations	Improper quality control	Failure to perform or defect in quality control of executive steps.
6		Ignore the symptoms	Signs of danger have been given but not considered.
7		False argument	The incorrect argument that leads to the accident.
8		Incorrect diagnosis/prediction	The main event has been predicted, but its side effects have been ignored.
9		Lack of access or defect in observations	Inability to access complete information for decision making.
10		Delayed interpretation	The interpretations required to make the decision have been delayed.
11		Failure to perform the necessary controls	Failure to perform the necessary and step controls.
12	Planning/processes	Improper design	Choosing the wrong design according to the current situation.
13		Prioritization/scheduling error	Wrong prioritization in planning.
14		Improper construction method	The selected method is inappropriate.
15	Equipment	Equipment failure	Failure to perform timely repairs and maintenance.
16		Software error	Switching off the warning or error reporting systems.
17		Equipment deduction	Lack of proper equipment to perform executive operations or their wear.
18	Organizing	Improper chart	The organizational chart is inappropriate for the type of project.
19		Assigning inappropriate tasks	Assigning wrong or incomplete tasks.
20		Absence of an HSE safety officer	Absence of the HSE officer on the worksite during the operation.
21		Absence of workshop supervisor	Absence of the worksite supervisor during the operation.
22		Lack of training	The workforce is not professionally trained.
23		Improper working hours	Performing operations at inappropriate hours.
24	Individual activities	Physical defects	Occupational medicine is not done for the workforce and the worker does not have a work permit.
25		Fear-stress	Fear or stress in performing executive operations.
26		Distractions	The desired force is forgetful.
27		Carelessness	Jokes or the like.
28		Variety of work	Performing various tasks with a limited number of personnel.
29		Fatigue	Incompatibility of the duration of work with the type of work.
30		Improper learning	The inability of the force to learn.
31	Environmental conditions	Improper temperature	Inadequate air temperature during the operation.
32		Improper sound	Inadequate noise or error signals.
33		Inadequate humidity	Inadequate air humidity during operations.
34		Inadequate lighting	Inadequate lighting during executive operations.
35	Relief and secure	Failure to implement fire alarm system	Implementation of a fire alarm system in the place of storage of incendiary cases.
36		Lack of firefighting	Deployment of firefighting less than 5 min from the project site.
37		Lack of emergency medical teams	Deployment of relief teams less than 5 min from the project site.
38		Lack of safety equipment	Deployment of safety equipment required in the project by the type of executive operations.
39	Technology	Excessive reliance on technology	Given the lack of development of artificial intelligence and the reliability of technology, the system should not be left alone.
40		Technology does not conform to existing conditions	Using technology in similar processes regardless of available variables.
41		Lack of familiarity with technology	Lack of familiarity with technology both in choosing and managing it.

**Table 3:** Prioritization of human error indices by the SWARA method.

No.	Group	Factor (Code)	Mean Relative Importance	*Kj*	Initial Weight	Normal Weight
1	Wrong actions	Time (C_1_)	1	1	1	0.1226
2	Observations/interpretations	Delayed interpretation (C_10_)	0.136	1.163	0.88	0.1080
3	Observations/interpretations	Misdiagnosis/prediction (C_8_)	0.090	1.090	0.81	0.0990
4	Wrong actions	Instrumental (C_3_)	0.146	1.146	0.70	0.0864
5	Observations/interpretations	Failure to perform necessary controls (C_11_)	0.052	1.052	0.67	0.0821
6	Wrong actions	Functional (C_2_)	0.299	1.299	0.52	0.0632
7	Planning/scheduling	Prioritization/scheduling error (C_13_)	0.114	1.114	0.46	0.0567
8	Equipment	Equipment fraction (C_17_)	0.125	1.125	0.41	0.0504
9	Equipment	Software error (C_16_)	0.900	1.090	0.38	0.0463
10	Organizing	Lack of training (C_22_)	0.107	1.107	0.34	0.0418
11	Individual activities	Fatigue (C_29_)	0.217	1.217	0.28	0.0343
12	Organizing	Improper chart (C_18_)	0.183	1.183	0.24	0.0290
13	Equipment	Equipment failure (C_15_)	0.253	1.253	0.19	0.0232
14	Organizing	Improper working hours (C_23_)	0.177	1.177	0.16	0.0197
15	Wrong actions	Spatial (C_4_)	0.287	1.287	0.12	0.0146
16	Planning/processes	Improper design (C_12_)	0.051	1.051	0.12	0.0146
17	Environmental conditions	Inadequate humidity (C_33_)	0.102	1.102	0.11	0.132
18	Planning/processes	Improper construction method (C_14_)	0.207	1.207	0.09	0.0109
19	Organize	Absence of safety officer (C_20_)	0.078	1.078	0.08	0.0102
20	Individual activities	Variety of work (C_28_)	0.050	1.050	0.08	0.0097
21	Relief and rescue	No firefighting deployment (C_36_)	0.115	1.115	0.07	0.0087
22	Individual activities	Carelessness (C_27_)	0.124	1.124	0.06	0.0077
23	Observations/interpretations	Lack of access or defect in observations (C_9_)	0.225	1.225	0.05	0.0063
24	Technology	Technology mismatch with existing conditions (C_40_)	0.320	1.320	0.04	0.0048
25	Environmental conditions	Inadequate lighting (C_34_)	0.071	1.071	0.04	0.0045
26	Observations/interpretations	Ignoring symptoms (C_6_)	0.033	1.033	0.04	0.0043
27	Observations/interpretations	Improper quality control (C_5_)	0.099	1.099	0.03	0.0039
28	Individual activities	Fear-stress (C_25_)	0.221	1.221	0.03	0.0032
29	Individual activities	Physical disability (C24)	0.058	1.058	0.02	0.0030
30	Technology	Excessive reliance on technology (C_39_)	0.058	1.058	0.02	0.0029
31	Environmental conditions	Bad sound (C_32_)	0.162	1.162	0.02	0.0025
32	Individual activities	Distraction (C_26_)	0.297	1.297	0.02	0.0019
33	Organizing	Assignment of improper tasks (C_19_)	0.047	1.047	0.01	0.0018
34	Organizing	Absence of workshop supervisor (C_21_)	0.198	1.198	0.01	0.0015
35	Rescue and rescue	Non-deployment of emergency medical teams (C_37_)	0.251	1.251	0.01	0.0012
36	Technology	Lack of familiarity with technology (C_41_)	0.114	1.114	0.01	0.0011
37	Environmental conditions	Inadequate temperature (C_31_)	0.056	1.056	0.01	0.0010
38	Relief and rescue	Lack of safety equipment (C_38_)	0.088	1.088	0.01	0.0009
39	Observations/interpretations	False argument (C_7_)	0.043	1.043	0.01	0.0009
40	Individual activities	Inadequate learning (C_30_)	0.306	1.306	0.01	0.0007
41	Relief and rescue	Failure of the fire alarm system (C_35_)	0.182	1.182	0.00	0.0006

## Data Availability

Not applicable.
